# DNA vaccines targeting the encoded antigens to dendritic cells induce potent antitumor immunity in mice

**DOI:** 10.1186/1471-2172-14-39

**Published:** 2013-08-14

**Authors:** Jun Cao, Yiqi Jin, Wei Li, Bin Zhang, Yang He, Hongqiang Liu, Ning Xia, Huafeng Wei, Jian Yan

**Affiliations:** 1 Interventional Oncology, Dahua Hospital, Xuhui District, Shanghai 200237, China; 2 Interventional Radiology, Suzhou Municipal Hospital, Suzhou, Jiangsu Province 215002, China; 3 Department of Geriatric Neurology, Brain Hospital Affiliated to Nanjing Medical University, Nanjing 210029, China; 4 Department of Institution of Hepatobililary and Gastrointestinal Diseases, Second Artillary General Hospital, Beijing 100088, China; 5 International Joint Cancer Institute, Second Military Medical University, Shanghai 200433, China

**Keywords:** DNA vaccine, DC-targeted, HER2/neu, Breast cancer, Cyclophosphamide

## Abstract

**Background:**

Although DNA vaccine holds a great potential for cancer immunotherapy, effective long-lasting antitumoral immunity sufficient to induce durable responses in cancer patients remains to be achieved. Considering the pivotal role of dendritic cells (DC) in the antigen processing and presentation, we prepared DC-targeting DNA vaccines by fusing tumor-associated antigen HER2/neu ectodomain to single chain antibody fragment (scFv) from NLDC-145 antibody specific for DC-restricted surface molecule DEC-205 (scFv^NLDC-145^), and explored its antitumoral efficacy and underlying mechanisms in mouse breast cancer models.

**Results:**

In vivo targeting assay demonstrated that scFv^NLDC-145^ specifically delivered DNA vaccine-encoded antigen to DC. Compared with untargeted HER2/neu DNA vaccines, vaccination with scFv^NLDC-145^-HER2/neu markedly promoted the HER2/neu-specific cellular and humoral immune responses with long-lasting immune memory, resulting in effective protection against challenge of HER2/neu-positive D2F2/E2 breast tumor while ineffective in parental HER2/neu-negative D2F2 breast tumor. More importantly, in combination with temporary depletion of regulatory T cells (Treg) by low-dose cyclophosphamide, vaccination with scFv^NLDC-145^-HER2/neu induced the regression of established D2F2/E2 breast tumor and significantly retarded the development of spontaneous mammary carcinomas in transgenic BALB-neuT mice.

**Conclusion:**

Our findings demonstrate that DC-targeted DNA vaccines for in vivo direct delivery of tumor antigens to DC could induce potent antigen-specific cellular and humoral immune responses and, if additional combination with systemic Treg depletion, was able to elicit an impressively therapeutic antitumoral activity, providing a rationale for further development of this approach for cancer treatment.

## Background

During the last 20 years, DNA-based immunization has been rapidly developed as a new approach to prime specific cellular and humoral immune responses to protein antigens [1]. In mouse models, DNA vaccines have been successfully directed against many types of tumor with tumor protection reproducibly observed in an antigen-specific manner [2]. However, conventional DNA vaccines with only the encoded antigen failed to mount an effective T-cell immunity in human trials, even when delivered by in vivo electroporation, which calls for a novel design for DNA vaccine [1,2].

It is critical for DNA vaccination to be successful that encoded proteins are taken up, processed, and presented by dendritic cells (DC), the most potent antigen-presenting cells (APC) *in vivo* that initiate the adaptive immunity. Following intradermal or intramuscular injection of a plasmid DNA vaccine in mice, the encoded gene is expressed in transfected keratinocytes and myocytes at the site of injection [3] as well as a small number of DC [4-6]. Keratinocytes and myocytes are poorly effective in presenting antigen and priming naive immune cells due to lack of expression of MHC class II and costimulatory molecules, and do not have ready access to T cells in lymphoid tissues, as is the case for DC [7]. It is thought that transduced DC initiate immune priming process, which can be further boosted by antigen released from other long-lived transfected cells [8,9]. Therefore, targeting DNA vaccines to DC should improve the efficacy of DNA vaccines. In fact, a recent study demonstrated that DC-targeted DNA vaccines elicited much higher level of antibody and antigen-specific T cells, leading to effective protection against virus expressing encoded antigen [10].

Coupling of antigens to ligands or antibodies that specifically bind to DC receptors has been widely used as a means of DC targeting [11]. Using this approach, a lowered requirement for antigen dose in stimulating immune responses in mice has been observed after targeting a variety of molecules, including MHC class II, DEC205, CD11c, Dectin-1/2, mannose receptor, and CD36 [12-17]. The studies have also shown that antibodies specific for the mannose receptor or DC-SIGN could effectively deliver antigen to human DCs, indicating that this strategy may also be applicable to human vaccination [18,19].

Overexpression of the HER-2 receptor tyrosine kinase has been found in various human malignancies, including breast, ovarian and gastric carcinomas, non-small cell lung cancer, and salivary gland cancers, and has been associated with poor prognosis of patients [20,21]. Endogenous HER2-specific CD4^+^ T cells and antibodies have been detected in patients with HER2-expressing cancers [22,23], and in clinical trials, HER2-specific CD4^+^ and CD8^+^ T-cell responses could be induced by peptide vaccination [24,25]. These studies provide strong supports for HER2 being an important tumor antigen for targeted immunotherapy. The clinically approved HER2-targeted immunotherapy involves infusion of humanized HER2-specific monoclonal antibody Herceptin; ref. [26]. Although Herceptin has been shown to be effective in inhibiting tumor growth in a limited population of HER2-postive metastatic breast cancer patients, elicitation of an active and more comprehensive immune response that includes both antibody and T-cell responses may provide more effective protection [27].

Here, we prepared DC-targeting DNA vaccines by fusing tumor-associated antigen HER2/neu ectodomain (HER2/neu, residues 22 to 561 or 22 to 582) to single chain antibody fragment (scFv) from NLDC-145 (scFv^NLDC-145^), a monoclonal antibody binding the mouse DC-restricted surface molecule DEC-205, and evaluated the preventive and therapeutic effects of these DNA vaccines in HER2/neu-positive mouse breast tumor models. We further characterized the cellular mechanisms driving antitumor effect of DC-targeted DNA vaccines elucidating the basic processes necessary to achieve immune-mediated tumor rejection.

## Methods

### Mice and cell lines

Six to 8-week-old female BALB/c (H-2^d^) mice were purchased from the Animal Experimental Center of the Second Military Medical University. BALB-neuT mice (H-2^d^) expressing a transforming neu under the control of mouse mammary tumor virus promoter were obtained from Charles River Laboratories (Shanghai, China). Heterozygous 6- to 15-week-old virgin females expressing rat neu as verified by PCR were used throughout this work. All animal studies were approved by the Institutional Review Board of the Second Military Medical University, Shanghai, China.

Mouse thymoma cell line EL4, breast cancer cell line 4 T1, and 293 T cell line were purchased from ATCC (American Type Culture Collection, VA, USA). The cells were maintained in DMEM supplemented with 10% FCS, 4 mmol/L glutamine, 100 units/mL penicillin and 100 μg/mL streptomycin. D2F2/E2 and EL4/E2 stably expressing human wild-type HER2 were maintained in complete DMEM medium containing 0.4 mg/mL G418 (Sigma-Aldrich). TUBO cells are neu-expressing breast carcinoma cells established from a lobular carcinoma of a female BALB-neuT mouse [28], and maintained in DMEM containing 20% FCS. Murine lymphocytes were cultured in RPMI-1640 containing 10% FCS, 2 mmol/L glutamine and 50 μmol/L 2-mercaptoethanol. All tissue culture reagents were purchased from Life Technologies unless described otherwise.

### Reagents

Peptides used in this study were obtained from Sigma-Aldrich. All peptides were > 95% pure as indicated by analytical HPLC. Lyophilized peptides were diluted in DMSO and stored at - 20°C until use. Recombinant HER2 and TRP2 protein were purchased from R&D Systems. Cyclophosphamide (CTX) were obtained from Sigma-Aldrich and reconstituted in sterile PBS (20 mg/mL) for in vivo injections. Monoclonal antibodies (mAbs) to the following antigens were purchased from eBiosciences (San Diego, CA): CD4 (GK 1.5) and CD8 (53–6.7) conjugated to fluorescein isothiocyanate (FITC); CD11c (N418) and Foxp3 (FJK-16 s) conjugated to phycoerythrin (PE); mAbs to PE-TNF-α (MP6-XT22) and PE-IFN-γ (XMG1.2) were purchased from BD PharMingen (San Jose, CA). Immunoglobulins with isotypes corresponding to the above mAbs and conjugated to the appropriate fluorochromes, were used as control for nonspecific binding.

### Construction of DNA vaccines

The backbone for the construction of DNA vaccines was the mammalian expression vector pcDNA3.1 (Invitrogen). In this vector encoding vaccine proteins are expressed under the control of the CMV promoter as an in-frame fusion with an antibody kappa chain signal peptide (SP) sequence (amino acid MDFQVQIFSFLLISASVIISRG) for secretion and are followed by C-terminal His tag for detection. The genes encoding the variable regions of the heavy (VH) and light (VL) chains of scFv^NLDC-145^ were synthesized according to the published sequences [29]. Each VH fragment was bound to its VL partner by use of a spacer encoding a 15 amino-acid flexible linker (Gly_4_Sert)_3_, yielding scFv constructs scFv^NLDC-145^. The sequence encoding for the extracellular domain of human HER2 or its rat homologue neu was amplified from cDNA of SK-BR-3 and TUBO cell lines using the following primers HER2-HindIII-s 5′-TTAAGCTTAG CACCCAAGTGTGCACCGGCAC-3′, HER2-XbaI-as 5′-TTTCTAGACAAACAGTGCCTGGCATT CACATAC-3′ and neu-HindIII-s 5′-TTAAGCTTGGAGCCGCGGGTACCCAAGTGTG-3′, neu-XbaI -as 5′-TTTCTAGATCCAAAGCAGGTCTCTGAGCTGTTTTGAG-3′.The resultant encoding sequences were then cloned in-frame downstream of the scFv^NLDC-145^. For in vivo targeting assay, we generated pcDNA3.1-scFv^NLDC-145^-EGFP by replacing the HER2 fragment with EGFP sequence cloned from pEGFP-N1 plasmid. The pcDNA3.1 vector encoding EGFP without DC-targeting scFv fragments as controls (pcDNA3.1 and pcDNA3.1-EGFP).

### Expression of protein encoded by DNA vaccines

The different pcDNA3.1 constructs were transiently transfected in 293T cells using Lipofectamine 2000 according to the manual instruction (Invitrogen). The resultant supernatants were harvested at 72 hours post-transfection and concentrated and dialyzed using centrifugal filter devices (Amicon Ultra, 10K, millipore). Protein expression was analyzed by Western blotting using recombinant anti-His mAb (ab1187, Abcam).

### In vivo targeting assay

The 50 μg pcDNA3.1-scFv^NLDC-145^-EGFP and pcDNA3.1-EGFP plasmids in 50 μL PBS were injected into the upper leg muscle of the left hind limb of the mice followed by in vivo electroporation as described previously [30]. One day later, the lysate of the muscle tissues of injection site were prepared using RIPA buffer and subjected to the western blotting for detection of EGFP and scFv^NLDC-145^-EGFP fusion protein by anti-EGFP antibody (ab111258, Abcam) as described above. The spleens were harvested from the injected mice at different time points (48, 60, 72 hours) and single-cell suspensions were prepared and stained with PE-conjugated anti-CD11c antibody or isotype control for 30 min. The GFP fluorescence in the CD11c-positive DC was analyzed by flow cytometry as described previously [14].

### Protective and therapeutic vaccination

For prophylactic vaccination, female BALB/c mice or BALB-neuT mice were vaccinated on days -21 and -7 by intramuscular injections of 50 μg pcDNA3.1-scFv^NLDC-145^-HER2/neu, pcDNA3.1-HER2/neu plasmid DNA in 50 μL PBS as described above. As control, 50 μL pcDNA3.1 or 50 μL PBS were injected. On day 0, animals were inoculated subcutaneously (s.c.) with 2 × 10^5^ D2F2/E2, D2F2 or TUBO tumor cells in the opposite flank. Tumor growth was monitored with a caliper by measuring two perpendicular tumor diameters every week, and tumor volumes were calculated according to the formula: length × (width)^2^ × 0.5. For therapeutic vaccination, when the tumors were 3–4 mm in diameter (day 7), mice were injected intraperitoneally (i.p.) with cyclophosphamide (100 mg/kg) in 100 μL PBS. Four days later (day 11), animals were vaccinated as described above. Vaccination was repeated once 14 days later, and tumor growth was followed. If animals appeared moribund or the diameter of the tumors reached 15 mm, the mice were sacrificed and this was recorded as the date of death for survival studies. For rechallenging experiments, the long-term surviving mice were injected s.c. either with 2 ×10^5^ D2F2/E2, D2F2, or 4T1 tumor cells.

### Prevention of spontaneous tumors

Preventive effects of the DNA vaccines were investigated in virgin female BALB-neuT mice that endogenously express neu in their mammary glands and develop neu-driven mammary carcinomas [28]. Animals were immunized twice at 8 and 10 week ages as described above. Mammary glands were inspected every week to monitor the appearance of tumors. Measurable/palpable masses >2 mm in diameter were regarded as tumors. Data are reported as tumor multiplicity (cumulative number of tumors per number of mice in each group) and shown as mean ±SE.

### Cytometric identification of regulatory T cells

For detection of regulatory T cells (Treg), splenocytes from immunized mice were surface stained with FITC anti-mouse CD4 (GK1.5; eBioscience). After that, cells were washed with wash buffer (PBS with 1% fetal bovine serum and 0.09% sodium azide), fixed and permeabilized with the Cytofix/Cytoperm reagent (BD Bioscience) for 20 minutes at 4°C, after which they were washed in Perm/Wash buffer (BD Bioscience), and stained with PE anti-mouse Foxp3 (FJK-16 s; eBioscience) at 4°C for 30 minutes. Immunoglobulin G-PE and immunoglobulin G-FITC (mouse) were used as negative controls. All analysis was performed on the FACSCalibur (Becton Dickinson) flow cytometer.

### Evaluation of T-cell responses

For detection of HER2-specific cellular immune response, splenocytes from vaccinated mice were cultured in 96-well flat-bottomed plates in 100 μL of growth medium in the presence of 10 μg/mL recombinant HER2 or TRP2 protein in vitro. After 80 h, [^3^H] thymidine (1 μCi/well; Amersham) was added for the remaining 16 h of the assay. [^3^H] thymidine incorporation was analyzed by liquid scintillation counting as described previously [14]. The supernatants were also collected and assayed for production of IFN-γ, TNF-α, IL-4, IL-10 by ELISA kits (R&D Systems).

For detection of IFN-γ and TNF-α-producing CD4 or CD8 T cells, intracellular cytokine staining assays were performed. Briefly, splenocytes harvested from vaccinated mice were cultured in the presence of 10 μg/mL recombinant HER2 or TRP2 protein for 24 h. During the final 4 h of incubation, 10 μg/mL brefeldin A were added. After surface staining with FITC-CD4 and FITC-CD8, cells were permeabilized and stained with PE-IFN-γ and PE-TNF-α prior to analysis by flow cytometry as described above. For CTL measurements, ^51^Cr-release assays were performed as described previously [14].

### Analysis of antibody responses

Peripheral blood was collected from the tail vein of the mice, and 1:100 dilutions of sera were analyzed by ELISA with recombinant HER2 protein or by flow cytometry using D2F2/E2 and TUBO tumor cells as described previously [14]. Normal mouse serum served as negative control.

### Statistical analysis

Differences among tumor growth kinetics, tumor multiplicity, and specific cytotoxicity were evaluated by ANOVA or the Student’s test. Values of P < 0.05 were considered significant. For survival studies, Kaplan-Meier survival curves were plotted and analyzed using Prism 5.00 software (GraphPad Software).

## Results

### Construction and expression of DNA vaccines

**Figure 1:**
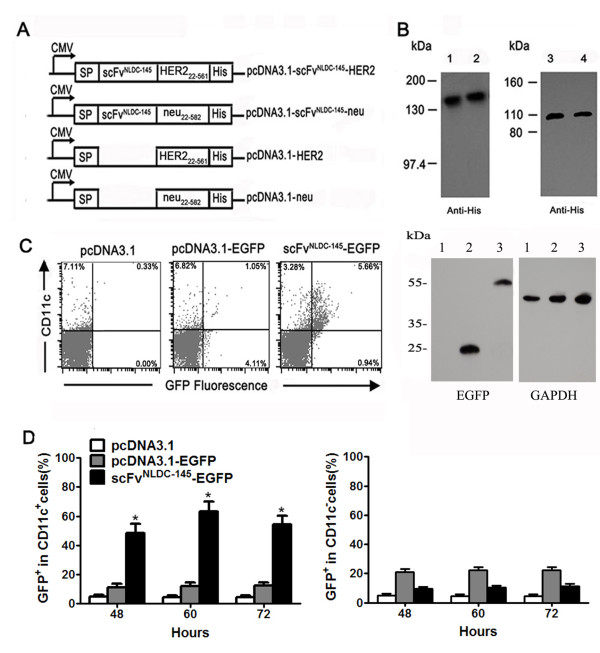
**scFv**^**NLDC-145 **^**targets tumor antigen into DCs in vivo. A** schematic representation of expression vectors. scFv^NLDC-145^-HER2, scFv^NLDC-145^-neu, pcDNA3.1-HER2, or pcDNA3.1-neu encode under the control of a CMV promoter, all the fusion proteins consisting of an signal peptide, amino acid residues 22 to 561 of human HER2 or amino acid residues 22 to 582 of rat neu, and COOH-terminal His tag. The control plasmids pcDNA3.1-HER2 and pcDNA3.1-neu lack the NLDC-145 domains. **B** 293T cells grown in 100-mm dishes were transfected with various expression vectors using Lipofectamine 2000 (invitrogen). Immunoblot analysis of supernatants from 293T cells transfected with scFv^NLDC-145^-HER2, scFv^NLDC-145^-neu, pcDNA3.1-HER2 and pcDNA3.1-neu (lane 1, 2, 3 and 4). Vaccine proteins were probed with mouse anti-His tag mAb followed by HRP-conjugated secondary anti-mouse antibody. **C left,** scFv^NLDC-145^-EGFP plasmid and controls as indicated were injected i.m. into mice, spleens were removed 48, 60, and 72 h thereafter, and stained with PE-conjugated anti-CD11c antibody. The GFP fluorescence in splenocytes was measured by flow cytometry. Representative results from one animal of each group 60 h post-injection. **right,** the expression of EGFP (MW,~25 kDa) and scFv^NLDC-145^-EGFP (MW,~55 kDa) fusion protein in injected muscle tissues detected by western blotting using anti- EGFP antibody 24 h after plasmid injection with GAPDH (MW,42 kDa) as loading control. Lane 1, mice treated with pcDNA3.1 control vaccine; lane 2, mice treated with EGFP-encoding vaccine; lane 3, mice treated with scFv^NLDC-145^-EGFP vaccine; **D** percentages of GFP^+^ DC in total DC (*left panel*); Percentages of GFP^+^ in CD11c-negative splenocytes (*right panel*). Bars, SE. *, P < 0.01, scFv^NLDC-145^-EGFP compared with other groups.

We obtained the genes encoding scFv^NLDC-145^ by whole gene synthesis according to the published sequences [29]. The carboxyl terminus of the scFv^NLDC-145^ was directly fused in-frame to the sequences encoding the extracellular domain of HER2 (amino acids 22–561) or neu (amino acids 22–582) amplified from SK-BR-3 or TUBO breast cell lines, followed by His tag (Figure 1A). To confirm the expression of these constructs, 293T cells were transiently transfected with these plasmids, and then supernatants were harvested 72 hours later and tested for protein secretion by Western blotting. As shown in Figure 1B, we detected the production of scFv^NLDC-145^-HER2 and scFv^NLDC-145^-neu proteins (lane 1, 2) or HER2 and neu fragments (lane 3, 4) in the supernatants by anti-His tag antibody respectively, and their molecular weights were slightly larger than predicted, indicating certain extent of glycosylation.

### scFv^NLDC-145^ targets antigen to DC in vivo

To test whether scFv^NLDC-145^ moiety delivery encoded antigens into DC in vivo, we constructed pcDNA3.1-scFv^NLDC-145^-EGFP and pcDNA3.1-EGFP plasmids (Additional file 1: Figure S1), which facilitated us to trace the final fate of antigens carried by scFv^NLDC-145^. The animals were injected i.m. with scFv^NLDC-145^-EGFP plasmids, sacrificed at different time points post-injection, and splenocytes were harvested and probed for GFP fluorescence by flow cytometry. We first confirmed the comparative expression of EGFP and scFv^NLDC-145^-EGFP fusion protein with correct molecular weight in the muscle tissue of injection site by western blotting (Figure 1C, right). As shown in Figure 1D, approximate 50% CD11c-positive DC in the spleen from pcDNA3.1-scFv^NLDC-145^-EGFP-treated animals showed GFP fluorescence at 48 h post-injection, and this percentage further went up, approaching about 65% at 60 h post-injection. Antigen loading was DC-specific since CD11c-negative non-DC cells showed few, if any, GFP fluorescence. A representative dot plot obtained at 60 h post-injection was shown in Figure 1C, left. In contrast, after injection of pcDNA3.1-EGFP plasmid, some fluorescence slightly above background levels were detectable, resulting in only 10% of DC being loaded (background level was 5%). The much lower percentage of GFP-positive DC in pcDNA3.1-EGFP-treated mice was not due to lower GFP expression efficiency of pcDNA3.1-EGFP plasmid since total GFP-positive cells in splenocytes were almost same from mice receiving injection of these two constructs.

### Protection of mice from challenge with HER2-expressing tumor cells

**Figure 2:**
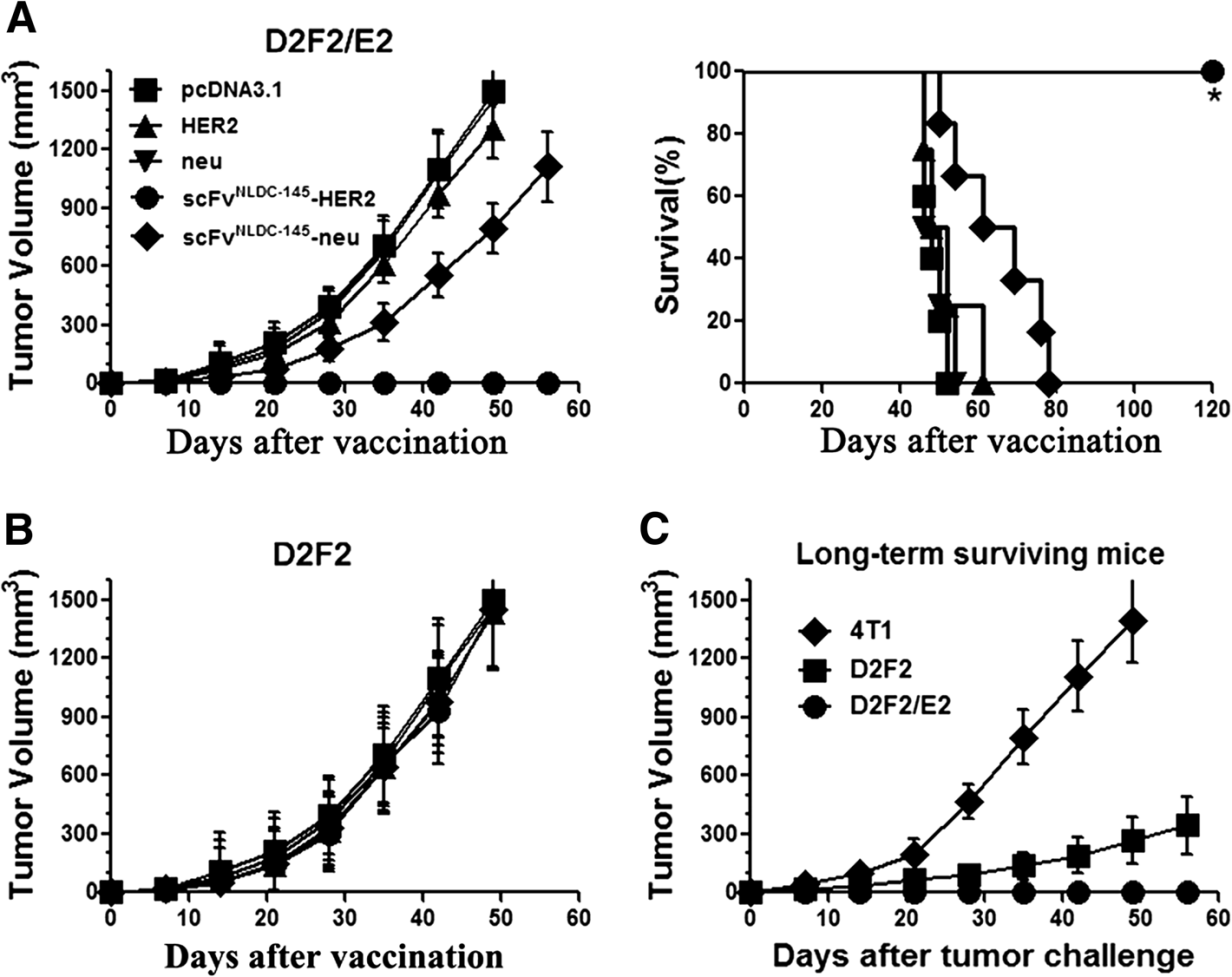
**Vaccination with scFv**^**NLDC-145**^**-HER2 protects mice from challenge with HER2-expressing tumor cells and induces memory immune responses. A** animals (10 mice per group) were vaccinated with HER2, neu, scFv^NLDC-145^-HER2 or scFv^NLDC-145^-neu on days -21 and -7. Control animals received pcDNA3.1. On day 0, mice were inoculated s.c. with D2F2/E2 tumor cells. Tumor developments were monitored, and animal survival was calculated. *Left panel*, kinetics of tumor growth; *Right panel*, survival curve. **B** the vaccinated animals were challenged with parental HER2-negative D2F2 cells on day 0. **C** the long-term surviving mice from scFv^NLDC-145^-HER2 group were rechallenged with D2F2/E2, D2F2, or syngeneic unrelated 4 T1 tumor cells 3 months after initial tumor challenge. Tumor growth was followed. Bars, SE. *, P < 0.001, scFv^NLDC-145^-HER2 compared with other groups.

To investigate whether immunization with DC-targeted vaccines induce antitumoral immunity and protect animals from subsequent tumor challenge, BALB/c mice were i.m. vaccinated twice at two week interval with various vaccines. Seven days after last immunization, the mice were subcutaneously challenged with HER2-positive D2F2/E2 tumor cells, and tumor development was monitored. As shown in Figure 2A, scFv^NLDC-145^-HER2 vaccination protected mice from D2F2/E2 tumor challenge, resulting in 100% survival in all mice during the observation period (120 days after vaccination). Furthermore, animals treated with scFv^NLDC-145^-neu exhibited slower tumor growth compared with those treated with untargeted HER2 or neu DNA vaccine or control pcDNA3.1, resulting in prolonged survival of these mice, which indicated the induction of cross-reactivity toward HER2 antigen by scFv^NLDC-145^-neu vaccination. To examine whether HER2-specific responses induced by scFv^NLDC-145^-HER2 vaccines were responsible for protection, a similar experiment was done using parental HER2-negative D2F2 tumor cells. Rapid tumor growth was observed in all animals regardless of either treatment (Figure 2B), suggesting that antitumor activity against HER2-expressing D2F2/E2 tumor cells was due to HER2-specific immune responses induced by scFv^NLDC-145^-HER2. We also obtained a similar protective effect of scFv^NLDC-145^-HER2 in C57BL/6 mice using paired EL/4 and EL4/E2 tumor models (data not shown).

To test whether immunologic memory was developed, long-term surviving mice initially vaccinated with scFv^NLDC-145^-HER2 were rechallenged with D2F2/E2 tumor cells. The parental D2F2 cells or unrelated syngeneic 4 T1 cells were used as controls. As shown in Figure 2C, the mice rejected subsequent rechallenges with the D2F2/E2 tumor cells and remained tumor-free, however, the mice could not reject unrelated syngeneic 4 T1 tumor. Interestingly, mice were also resistant to the rechallenge with the parental D2F2 tumor cells with 70% of mice tumor-free and remaining mice displaying drastically slow tumor growth. In summary, these results indicate that scFv^NLDC-145^-HER2 vaccination induced long-lasting HER2-specific antitumor immunity, which could protect mice from HER2-expressing tumor challenge.

### Induction of HER2-specific T cells

**Figure 3:**
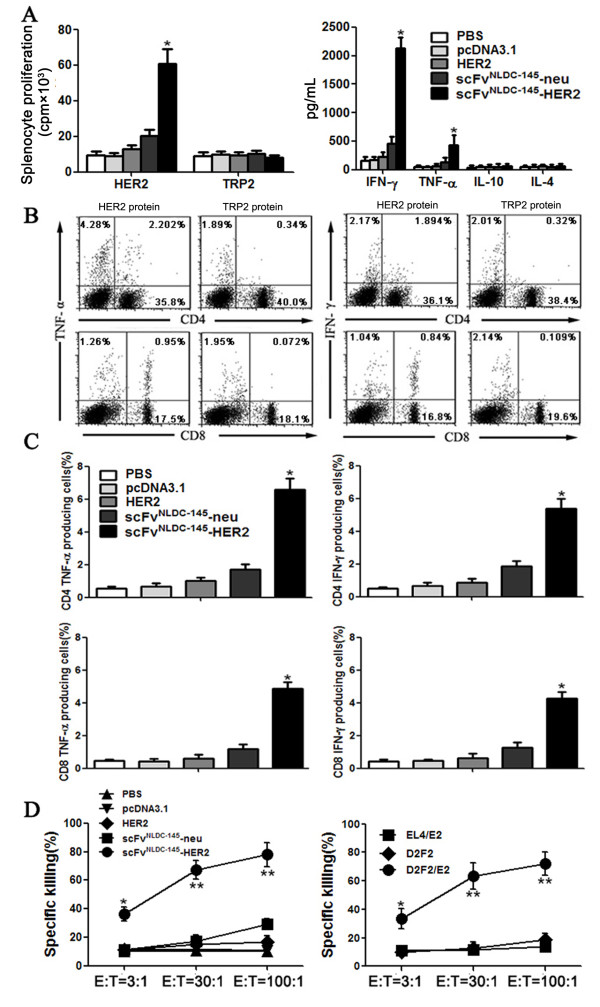
**Vaccination with scFv**^**NLDC-145**^**-HER2 induced HER2-specific cellular immune response.** BALB/c mice were vaccinated with HER2, scFv^NLDC-145^-neu or scFv^NLDC-145^-HER2. Control animals received PBS or pcDNA3.1. Spleens and peripheral blood were harvested from the vaccinated animals after two vaccinations. **A** splenocytes isolated from the vaccinated animals were cultured in the presence of 10 μg/mL recombinant HER2 or TRP2 protein for 4 d with the addition of [^3^H] thymidine in the last 16 h. T-cell proliferation was determined by [^3^H] thymidine incorporation (*left panel*). *Right panel*, the supernatant recovered from the assay in *left* was tested for cytokine production by ELISA. **B** intracellular staining for IFN-γ and TNF-α in splenocytes from the vaccinated animals stimulated with 10 μg/mL recombinant HER2 or TRP2 protein for 24 h and brefeldin A added during the final 4 h of incubation. One representative dot plots from scFv^NLDC-145^-HER2-vaccinated animal. **C** percentage of CD4^+^TNF-α^+^, CD4^+^IFN-γ^+^, CD8^+^ TNF-α^+^ and CD8^+^ IFN-γ^+^ cells in total CD4^+^ and CD 8^+^ T cells from each group are shown. The data are mean percentages ± SE. **D** splenocytes were cocultured with D2F2/E2 cells for 5 d. The resultant splenocytes (E) were cocultured for 4 h with the ^51^Cr-labeled target cells (T) (*left panel*). *Right panel*, the restimulated splenocytes from scFv^NLDC-145^-HER2 vaccinated mice were also cocultured for 4 h with the ^51^Cr-labeled D2F2 or EL4/E2 (different gene background control) target cells. Percentages of target cells killing by the splenocytes from the vaccinated mice are shown. Data represent the means of triplicate cultures and are representative of two independent experiments. Bars, SE. *, P < 0.01, **, P < 0.001, scFv^NLDC-145-^HER2 compared with other groups.

To analyze the nature of the immune responses induced by scFv^NLDC-145^-HER2, splenocytes were isolated from the vaccinated mice and cultured in the presence of recombinant HER2 or TRP2 protein for 3 days *in vitro*. As shown in Figure 3A, left, splenocytes obtained from scFv^NLDC-145^-HER2-vaccinated mice showed vigorous proliferation upon stimulation with recombinant HER2 protein, but not TRP2 protein. A slightly increased proliferation was also detected in the splenocytes from scFv^NLDC-145^-neu-vaccinated mice. In contrast, no evident T-cell proliferation could be observed when mice were vaccinated with untargeted HER2 or neu.

The supernatants of stimulated T cells were tested for the presence of cytokines by ELISA. Splenocytes obtained from scFv^NLDC-145^-HER2-vaccinated mice produced substantial amounts of TNF-α and IFN-γ (Figure 3A, right); similarly, a mildly higher level of IFN-γ and TNF-α cytokine was also detected in the supernatant from scFv^NLDC-145^-neu-vaccinated mice. We did not detect the secretion of IL-4 and IL-10 cytokines with immunosuppressive activity in all groups.

Next, we evaluated for the induction of HER2-specific CD4^+^ and CD8^+^ T cells. As shown in Figure 3C, a much higher percentage of CD4^+^ and CD8^+^ T cells producing IFN-γ and TNF-α was detected upon in vitro restimulation with recombinant HER2 protein in splenocytes from scFv^NLDC-145^-HER2-vaccinated mice. These T cells were HER2-specific since no cells produced these two cytokines upon restimulation with recombinant TRP2 pretein. A representative dot plot was shown in Figure 3B. In addition, splenocytes from scFv^NLDC-145^-HER2-vaccinated mice exhibited significantly higher target cell killing than did those from other group mice (Figure 3D, left). The specificity of the killing was validated by the inability of the splenocytes to kill parental D2F2 target cells and EL4/E2 target cells with a different H-2^b^ background (Figure 3D, right). The cytotoxic effect was mediated by CD8^+^ T cells, because the killing was inhibited by addition of anti-CD8, but not anti-CD4, antibody (data not shown).

### Induction of HER2-specific antibody

**Figure 4:**
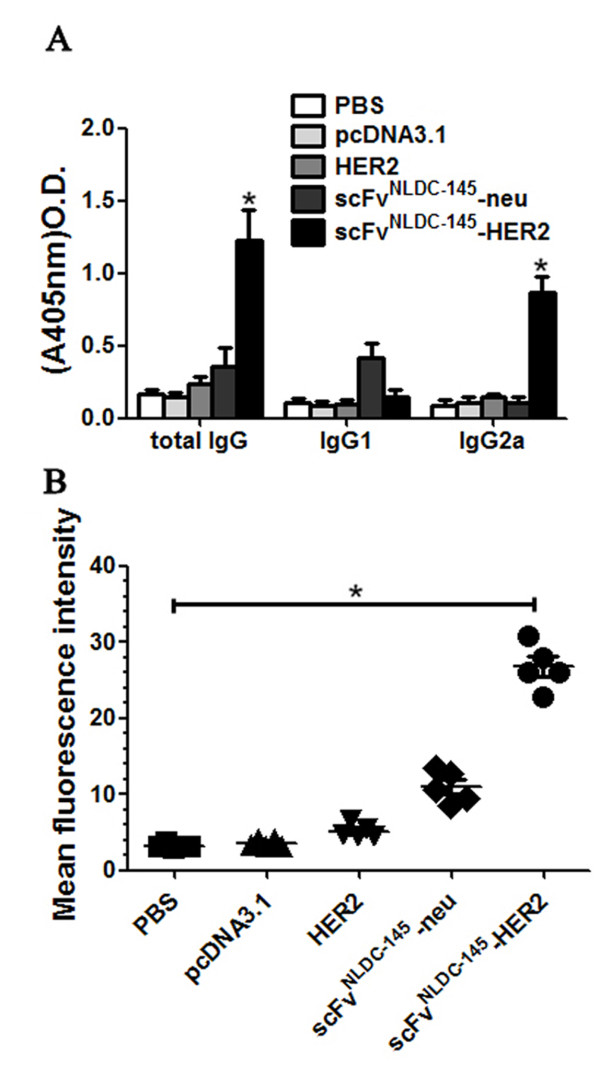
**Vaccination with scFv**^**NLDC-145**^**-HER2 induced HER2-specific antibodies.** BALB/c mice were vaccinated with HER2, scFv^NLDC-145^-neu or scFv^NLDC-145^-HER2. Control animals received PBS or pcDNA3.1. Sera were collected from the vaccinated animals after two vaccinations. **A** HER2-specific total IgG and IgG subclass (IgG1and IgG2a) antibodies in sera from the vaccinated animals after 1:100 dilution were determined by ELISA. The mean OD405 values of pooled sera from each group (5 mice per group) were presented. Bars, SE. The background OD405 of normal mouse sera was < 0.04. **B** D2F2/E2 cells were incubated with the immune sera obtained from the vaccinated animals, and surface bound antibodies were detected using FITC-labeled secondary antibodies. Samples were then analyzed by flow cytometry. The data were represented as mean fluorescence intensity. Normal mouse sera served as control. All results were representative of two independent experiments. *, P < 0.01, scFv^NLDC-145^-HER2 compared with other groups.

We also evaluated the induction of HER2-specific antibody. As shown in Figure 4A, vaccination with scFv^NLDC-145^-HER2 induced a significantly highly level of HER2-specific antibodies specifically binding to recombinant HER2 protein in ELISA assays. Detailed analysis of antibody isotype demonstrated that antibodies induced by scFv^NLDC-145^-HER2 vaccine was mainly IgG2a, which was consistent with cytokine profile of splenocytes from scFv^NLDC-145^-HER2-vaccinated mice (Figure 4A).

We also tested whether the sera of vaccinated mice contained antibodies binding to “natural” D2F2/E2 cells. As shown in Figure 4B, we detected D2F2/E2-specific antibodies in sera derived from mice vaccinated with scFv^NLDC-145^-HER2, whereas no antibodies were found in other groups. In summary, the results show that scFv^NLDC-145^-HER2-vaccinated mice developed antibodies that recognize epitopes expressed by D2F2/E2 cells and thus may also confer protection against tumor growth *in vivo*.

### Therapeutic efficacy of scFv^NLDC-145^-HER2 vaccine

**Figure 5:**
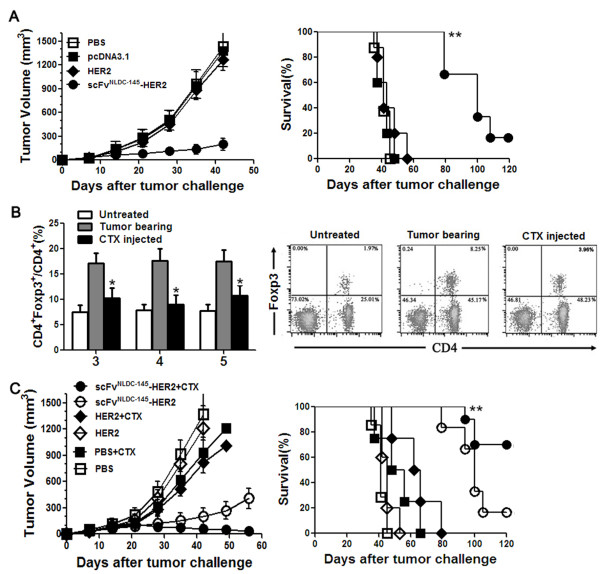
**Therapeutic efficacy of scFv**^**NLDC-145**^**-HER2 vaccine. A** BALB/c mice (10 mice per group) were inoculated s.c. with D2F2/E2 tumor cells. On day 7, animals with tumors sizing ~ 40 mm^3^ were immunized with scFv^NLDC-145^-HER2 or respective controls. Treatment was repeated on day 21. Tumor developments were monitored, and animal survival was calculated. *Left panel*, kinetics of tumor growth; *Right panel*, survival curve. The data were represented as the mean tumor volume (mm^3^) and representative of two experiments with comparable results. **, P < 0.01, scFv^NLDC-145^-HER2 compared with other groups. **B** temporary depletion of CD4^+^ Foxp3^+^ regulatory T cells by a single injection of low-dose CTX. BALB/c mice (3 mice per group) were inoculated s.c. with D2F2/E2 tumor cells. When the tumors were 3-4 mm in diameter (day 7), mice were injected i.p. with CTX (CTX injected) or PBS (Tumor bearing). Naive mice (Untreated) were used as control. The spleens were harvested at different time points and analyzed for the regulatory T cells. *Left panel,* Percentage of CD4^+^ Foxp3^+^ in total CD4^+^ cells; *Right panel,* a representative dot plot. Bars, SE. *, P < 0.05, CTX group compared with other groups. **C** BALB/c mice (10 mice per group) were inoculated s.c. with D2F2/E2 tumor cells. When the tumors were 3-4 mm in diameter (day 7), mice received CTX injection. Four days later (day 11), animals were vaccinated with various DNA vaccines. Treatment was repeated after two weeks. *Left panel,* kinetics of tumor growth; *Right panel,* survival curve. The data were represented as the mean tumor volume (mm^3^) and representative of two experiments with comparable results. **, P < 0.01, scFv^NLDC-145^-HER2/CTX compared with other groups.

We next evaluated the therapeutic effect of scFv^NLDC-145^-HER2 vaccination on established tumors in D2F2/E2 breast tumor model. BALB/c mice were subcutaneously inoculated with D2F2/E2 tumor cells. On day 7, animals with tumors sizing ~ 40 mm^3^ were randomized into groups treated with scFv^NLDC-145^-HER2 or respective controls. Treatment was repeated once 2 week later. As shown in Figure 5A, scFv^NLDC-145^-HER2 vaccination substantially slowed tumor development and protected up to 20% (2/10) of the mice from tumor growth at the end of experiment (120 days after tumor inoculation).

Since regulatory T cells (Treg) have been shown to mediate immune-tolerance towards tumor-antigens in various tumor models, we further tested whether systemic depletion of regulatory T cells would increase the therapeutic efficacy of DC-targeting vaccine. An approach with intraperitoneal injection of low-dose (100 mg/kg) cyclophosphamide (CTX) that was successfully applied in various models was utilized this study. As shown in Figure 5B, low-dose CTX injection in the D2F2/E2-bearing mice efficiently depleted Treg almost to the level in tumor-free mice 4 days post-injection, however, no direct killing effect on tumor cells were observed (data not shown). We therefore tested DC-targeted vaccines in combination with Treg depletion by low-dose CTX. As shown in Figure 5C, this combination significantly improved the therapeutic effects of scFv^NLDC-145^-HER2 vaccine; at the end of experiment, 80% (16/20) mice rejected the established tumor and remaining 4 (20%) mice had stably small tumors (~30 mm^3^). These tumor-free mice also rejected the rechallenges with the same tumor cells (date not shown). Untargeted DC vaccines failed to exert therapeutic effects although this vaccine in combination with CTX mildly delayed tumor growth. The experiment was repeated with similar results (date not shown). The data indicate that DC-targeting vaccines are able to mount an impressively therapeutic antitumor effect when in combination with systemic Treg depletion.

### Antitumoral activity of the scFv^NLDC-145^-neu DNA vaccine in immunotolerant BALB-neuT mice

Tumor models based on human HER2-expressing D2F2/E2 cells are useful to assess the basic functionality of cancer vaccines, however, such models do not fully reflect the situation of human cancer usually characterized by immunologic tolerance toward HER2. Therefore, we further tested DC-targeted DNA vaccines in female BALB-neuT mice that represent an immunotolerant model of spontaneous cancer [28]. In these experiments, we used scFv^NLDC-145^-neu instead of scFv^NLDC-145^-HER2 since the spontaneous tumors developed in BALB-neuT mice are driven by constitutively activated rat neu protein.

**Figure 6:**
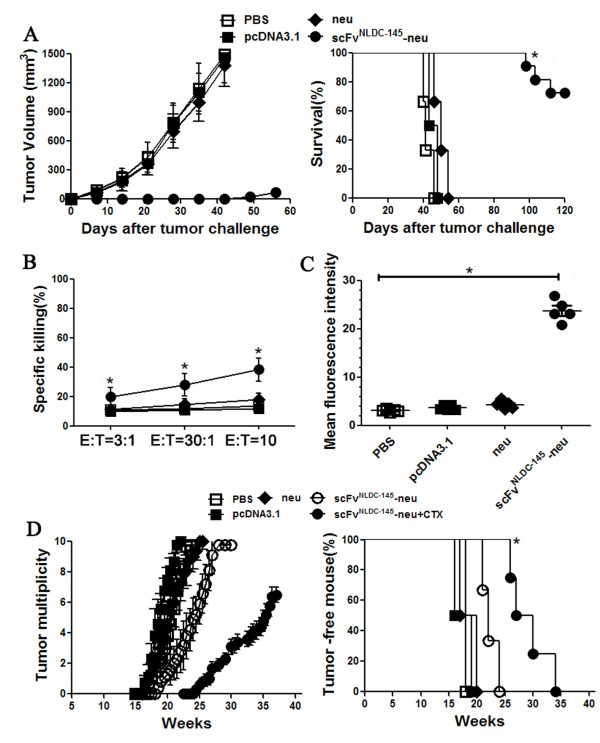
**Protective effects of scFv**^**NLDC-145**^**-neu in transgenic BALB-neuT mice. A** female BALB-neuT mice (10 mice per group) were vaccinated with neu or scFv^NLDC-145^-neu in left hind limb on days -21 and -7. Control animals received pcDNA3.1 or PBS. On day 0, mice were inoculated s.c. with neu-expressing TUBO cells in opposite flank. *Left panel,* kinetics of tumor growth; *Right panel,* survival curve. *, P < 0.01, scFv^NLDC-145^-neu compared with other groups. **B** in a separate experiment, splenocytes obtained from the vaccinated mice described above were tested for tumor killing activity. Data represented the means of triplicate cultures. *, P < 0.01, scFv^NLDC-145^-neu compared with other groups. **C** sera from the vaccinated mice described above were assayed for neu-specific antibodies by flow cytometry. The data were represented as mean fluorescence intensity. *, P < 0.01, scFv^NLDC-145^-neu compared with other groups. **D** animals were immunized neu or scFv^NLDC-145^-neu twice at weeks 8 and 10. One group mice with scFv^NLDC-145^-neu vaccination also received CTX injection 4 days before the first vaccination. Control animals received pcDNA3.1 or PBS. Development of mammary tumors was monitored by manual examination of the mammary glands once every week. Measurable masses of > 2 mm diameter were regarded as tumors. Points, mean number of tumors in each group (tumor multiplicity; *left panel*) and percentage of tumor-free mice (*right panel*); bars, SE. All results were representative of two to three independent experiments. *, P < 0.01, scFv^NLDC-145^-neu /CTX compared with other groups.

We first evaluated the preventive efficacy of scFv^NLDC-145^-neu vaccine using transplantable neu-expressing TUBO tumor model in BALB-neuT mice. BALB-neuT mice received twice scFv^NLDC-145^-neu or control vaccination at 14 days interval. One week after last vaccination, the animals were challenged with TUBO tumor cells. As shown in Figure 6A, the animals receiving scFv^NLDC-145^-neu vaccination were significantly protected against a subsequent challenge with TUBO cells. Sixty days after tumor challenge, 90% (9/10) mice in this group remained tumor free and 1 mouse had small tumors (~35 mm^3^). The experiment was repeated with similar results (data not shown). Mechanistic investigation showed that scFv^NLDC-145^-neu induced high levels of neu-specific CTLs and antibodies (Figure 6B,C).

The effect of scFv^NLDC-145^-neu vaccination in the prevention of spontaneous mammary tumors that naturally arise in BALB-neuT mice was also evaluated. The scFv^NLDC-145^-neu was given to the mice at week 8 from birth when diffuse atypical hyperplasia is already evident in the mammary glands but before in situ carcinoma is evident [31] and repeated at week 10. Mice in one group also received CTX injection 4 days before the first vaccination. As shown in Figure 6D, scFv^NLDC-145^-neu/CTX vaccination resulted in a significant prolongation of tumor-free survival. This corresponded with a marked delay (~4 weeks) in the appearance of macroscopically detectable tumors in the mammary glands of these mice. By week 38, all of the mice that were vaccinated with scFv^NLDC-145^-neu/CTX remained alive. In contrast, by week 26, all of the mice in the control groups had large tumors and required euthanasia. We again observed a mildly protective effect in scFv^NLDC-145^-neu vaccination group, in which the survival of most mice delayed to week 30.

## Discussion

Although clinical trials have shown that DNA vaccines could elicit immune responses in humans, the protective potency is modest [2]. The reasons for the failure of DNA vaccines to induce potent immune responses in humans have not been elucidated. It is reasonable to assume that low levels of antigen production, inefficient antigen presentation and insufficient stimulation of APC have roles in low potency of DNA vaccine [32].

Our results showed that scFv^NLDC-145^ could mediate antigen to be effectively phagocytosed by the DC compared with untargeted antigen *in vivo*. DEC205 expression is restricted to a subset of DC in mice that are specialized to cross-present exogenous antigens with resultant induction of MHC-I-restricted CD8^+^ CTLs and also promote the development of MHC-II-restricted CD4^+^ helper T cells [13,33]. Therefore, DEC205-carried antigens can be targeted to DEC205^+^ cross-presenting DC in the T-cell zone of spleen, which allows for antigen presentation by DC to both CD4^+^ and CD8^+^ T cells and provides a basis for the induction of a more powerful immune response compared with conventional vaccines. Confirming the pivotal role of CD8^+^ T cells, HER2-specific CD8^+^ CTLs specifically lysing HER2-expressing tumor cells were required to protect mice from HER2-positive tumor growth since antibody-mediated depletion of these cells abrogated the protective effects conferred by scFv^NLDC-145^-HER2 vaccine (Additional file 2: Figure S2), which is also consistent with the previous studies [34,35]. The scFv^NLDC-145^-HER2 vaccination induced high titer of HER2-specific IgG2a antibodies, indicating Th1-biased immune response. The data are also concordant with two recent studies showing vaccination with DNA vaccines encoding antigen fused to scFv^NLDC-145^ generated significantly stronger T-cell and antibody-specific responses compared with that elicited by untargeted vaccines [10,29].

The immune responses induced by scFv^NLDC-145^-HER2 vaccination were directed specifically against HER2 antigen as evidenced by protecting against HER2-positive D2F2/E2 but not parental HER2-negative D2F2 tumor cells in vaccinated animals. In addition, scFv^NLDC-145^-HER2 vaccination induced immunologic memory with vaccinated mice resistant to subsequent rechallenge with D2F2/E2 and D2F2 cells. Presumably, the development of long-term immunologic memory was not only dependent on the HER2 antigen but also on other unidentified antigens of D2F2/E2 tumor. Because antigen-negative variants may arise after antigen-positive tumor cells are destroyed, immune responses to additional undefined tumor-associated antigens may be crucial to the ultimate success of vaccination [14,31].

Vaccination with scFv^NLDC-145^-HER2 provided up to 20% of mice from HER2-positive tumor development in the therapeutic setting. The weakly therapeutic potency was possibly due to the insufficiency of local immune responses mounted by targeted DNA vaccine alone since pretreatment of mice with cyclophosphamide significantly increased the protective effects conferred by targeted vaccine with tumor regressed in 80% of mice. Low-dose CTX is known to selectively deplete Treg cells, with the nadir at day 4, and recovery to pretreatment levels by day 10 [36]. In addition, CTX has other immunomodulatory effects, including Th2/Th1 switch [37]; induction of type I IFN [38]; and the activation of DC [39]. It has also been reported that CTX pretreatment can remodel the local immune profile with increased IFN-γ-producing CD4^+^ and CD8^+^ effector T cells and decreased Treg cells in tumor microenvironment [40]. Consistent with previous studies [36,40], we observed that CTX treatment significantly decreased both peripheral (Figure 5B) and tumor-infiltrated Treg cells (data not shown), which may consequently promote local vaccine-induced CD8^+^ T-cell immune responses and induce more potent protection. It needs further investigation to clarify the correlation between local immune response and protective effects induced by targeted vaccine. Considering that reagents are readily available for depletion of Treg cells, our combined strategy holds potential for clinical translation.

Vaccination with scFv^NLDC-145^-neu significantly protected against a subsequent challenge with TUBO cells and combination with Tregs ablation markedly delayed the onset of spontaneous mammary carcinomas in BALB-neuT mice. The data were consistent with previous studies showing that Treg depletion enabled neu-specific CTL responses after vaccination of neu-transgenic FVB/N mice with a cellular vaccine expressing neu and granulocyte macrophage colony-stimulating factor [41] and was able to break tolerance to the immunodominant TYVPANASL epitope [42] and combined with peptide vaccination and adjuvants markedly extended disease-free survival in BALB-neuT mice [43]. Unlike protein-based DC-targeting vaccines, which require the co-injection of additional DC maturation stimuli to induce T cell responses [11,13,14], DC targeting in a DNA format induces immunity without additional adjuvant [28], possibly because the DNA itself provides some signals for DC maturation [44]. It warrants further study to determine whether the efficacy of DC-targeting vaccines could be further improved by additional stimuli that increase DC numbers and maturation state and/or improve the function of the responding T cells.

## Conclusion

In summary, our results show that DC-targeting DNA vaccines induce potent immune responses and provoke superior antitumoral effects in mouse breast tumor models; impressively, when combined with Treg depletion, DC-targeting DNA vaccines induce the regression of established tumor in transplantable tumors and significantly retard spontaneous tumor development in immunotolerant BALB-neuT mice. DEC205 is expressed at high levels on a large fraction of DCs in the T cell areas of lymphoid organs, including human lymphoid tissues [45], and this give perspective for its application in human. Since DC-targeting protein vaccines are being evaluated in clinical trials [46], it would be feasible to evaluate DC-targeting DNA vaccination in combination with Treg depletion for cancer treatment.

## Competing interests

The authors declare that they have no competing interests.

## Authors’ contributions

HFW and JY made substantial contributions to conception and design as well as to the interpretation of the data and drafted the manuscript. JC, YQJ and WL carried out the experiments. BZ, YH and HQL contributed to conception, the interpretation of the data and assisted to draft the manuscript. NX conceived of the study, participated in its design and coordination and helped to draft the manuscript. All authors read and approved the final manuscript.

## Supplementary Material

Additional file 1: Figure S1 Construction and expression of pcDNA3.1-scFv^NLDC-145^-EGFP and pcDNA3.1-EGFP **a** generated pcDNA3.1-scFv^NLDC-145^-EGFP by replacing the HER2 fragment with EGFP sequence cloned from pEGFP-N1 plasmid. The pcDNA3.1 vector encoding EGFP without DC-targeting scFv fragment as control. **b** 293T cells grown in 24-well plate were transfected with the two expression vectors using Lipofectamine 2000 (invitrogen). Green fluorescent protein GFP was observed by inverted fluorescence microscope (X51-A21PH, OLYMPUS). Click here for file

Additional file 2: Figure S2 Analysis of cell population responsible for the protective effects by targeted vaccine. Animals (5 mice per group) were vaccinated with scFv^NLDC-145^-HER2 on days -21 and -7. On day 0, mice were inoculated s.c. with D2F2/E2 tumor cells. For in vivo depletion of CD4^+^, CD8^+^ T cells or CD19^+^ B cells, an anti-CD4 (0.5 mg/mouse; Clone GK1.5), anti-CD8 (0.5 mg/mouse; Clone YTS 169.4), anti-CD19 (0.2 mg/mouse; Clone 1D3) or control (0.5 mg/mouse; Clone 2A3) mAb was injected i.p. on days -7, -3 and -1 and repeated weekly later. All mAbs were purchased from BioXcell. Tumor developments were monitored, and animal survival was calculated. Click here for file
