# Supplementary figures and images for: DNA vaccines targeting the encoded antigens to dendritic cells induce potent antitumor immunity in mice
Source: BMC Immunol. 2013 Aug 14;14:39. doi: 10.1186/1471-2172-14-39 (PMC3751307; doi:10.1186/1471-2172-14-39)

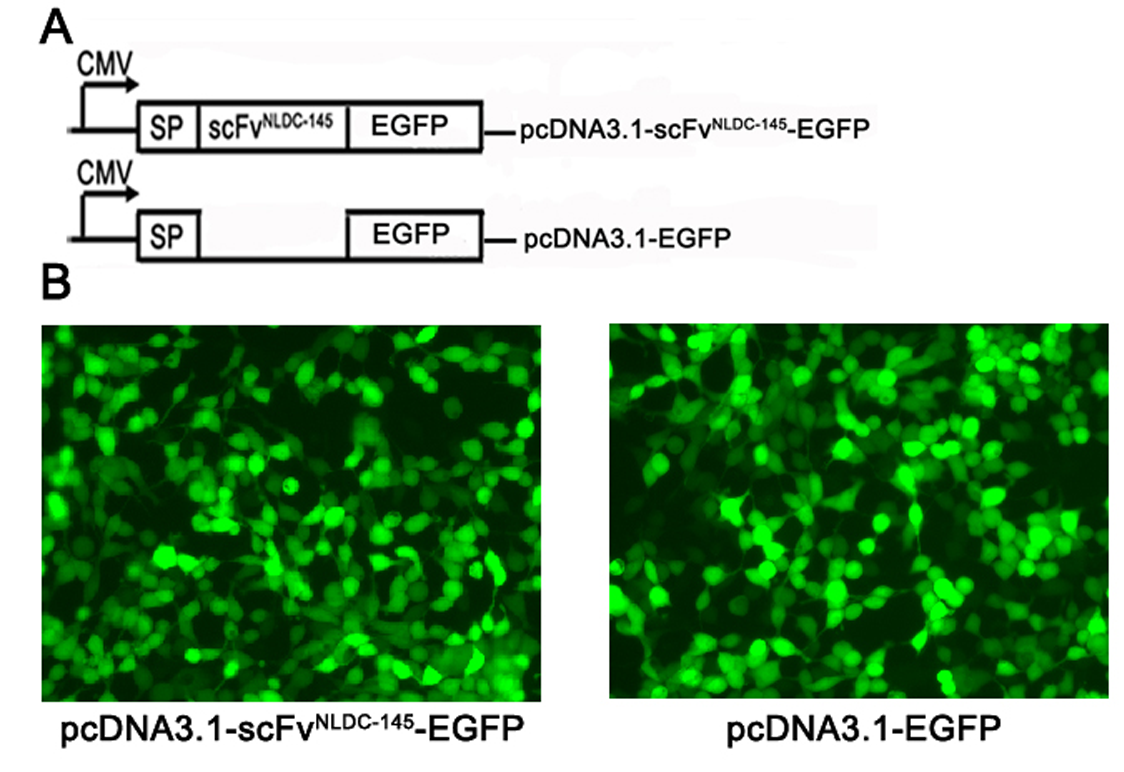

Supplement: Additional file 1: Figure S1 — Construction and expression of pcDNA3.1-scFvNLDC-145-EGFP and pcDNA3.1-EGFP a generated pcDNA3.1-scFvNLDC-145-EGFP by replacing the HER2 fragment with EGFP sequence cloned from pEGFP-N1 plasmid. The pcDNA3.1 vector encoding EGFP without DC-targeting scFv fragment as control. b 293T cells grown in 24-well plate were transfected with the two expression vectors using Lipofectamine 2000 (invitrogen). Green fluorescent protein GFP was observed by inverted fluorescence microscope (X51-A21PH, OLYMPUS). [file 1471-2172-14-39-S1.tiff]

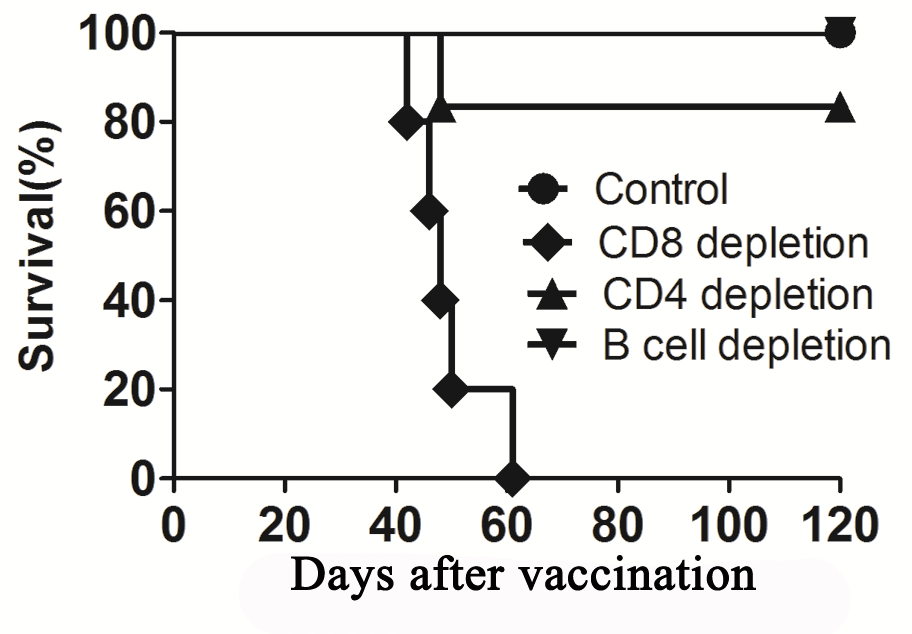

Supplement: Additional file 2: Figure S2 — Analysis of cell population responsible for the protective effects by targeted vaccine. Animals (5 mice per group) were vaccinated with scFvNLDC-145-HER2 on days -21 and -7. On day 0, mice were inoculated s.c. with D2F2/E2 tumor cells. For in vivo depletion of CD4+, CD8+ T cells or CD19+ B cells, an anti-CD4 (0.5 mg/mouse; Clone GK1.5), anti-CD8 (0.5 mg/mouse; Clone YTS 169.4), anti-CD19 (0.2 mg/mouse; Clone 1D3) or control (0.5 mg/mouse; Clone 2A3) mAb was injected i.p. on days -7, -3 and -1 and repeated weekly later. All mAbs were purchased from BioXcell. Tumor developments were monitored, and animal survival was calculated. [file 1471-2172-14-39-S2.tiff]
